# Consolidative thoracic radiotherapy for extensive stage small cell lung cancer

**DOI:** 10.18632/oncotarget.14759

**Published:** 2017-01-19

**Authors:** Xiaoli Zhang, Jinming Yu, Hui Zhu, Xue Meng, Minghuan LI, Liyang Jiang, Xingchen Ding, Xindong Sun

**Affiliations:** ^1^ Department of Oncology, Renmin Hospital of Wuhan University, Wuhan, China; ^2^ Department of Radiation Oncology, Shandong Cancer Hospital Affiliated to Shandong University, Shandong Academy of Medical Science, Jinan, China; ^3^ Shandong Academy of Medical Sciences, Jinan University, Jinan, China; ^4^ Weifang Medical University, Weifang, China

**Keywords:** small cell lung cancer, extensive stage, thoracic radiotherapy

## Abstract

Extensive stage small cell lung cancer (ES-SCLC) represents approximately half of all diagnosed small cell lung cancer worldwide. It is notorious for a high risk of local recurrence although it’s sensitive to chemotherapy. Nearly 90% of intrathoracic failures happen in the first year after diagnosis. The cornerstone of treatment for ES-SCLC is etoposide-platinum based chemotherapy. Consolidative radiotherapy to thorax has diminished the incidence of local relapse, therefore it should be offered to patients with excellent response to induction first-line chemotherapy. This review centers on the clinical evidence for the use of thoracic radiotherapy (TRT) and current modalities of TRT delivery, then tries to determine a feasible way to conduct TRT in a selective group of cases.

## INTRODUCTION

More than half of patients with newly diagnosed small cell lung cancer (SCLC) present with extensive stage (ES) disease, commonly defined by the Veterans Administration Lung Study Group (VALSG) staging system as disease which extends beyond the confines of the hemithorax, mediastinum, and ipsilateral or contralateral supraclavicular nodes and cannot be fully encompassed within a tolerable single radiotherapy treatment field [1].

The cornerstone of therapy for extensive stage small cell lung cancer (ES-SCLC) without brain metastases is platinum-based chemotherapy, which results in a median survival of approximately 9 to 12 months [2]. Although ES-SCLC is highly sensitive to chemotherapy, nearly all patients eventually experience relapse of disease, and only 5% of patients are alive 2 years after diagnosis [3]. More intensive chemotherapy strategies, molecularly targeted drugs, or maintenance chemotherapy have not appreciably prolonged survival period [4–9]. Given the radiosensitive nature of SCLC, radiotherapy directed at the brain, thorax, hemibody, or total body have been used in an attempt to improve overall survival (OS) [10–12]. Thoracic tumor progression is a major cause of morbidity for patients with ES-SCLC. Even after chemotherapy, 75% to 90% of patients have residual intrathoracic disease, and approximately 90% develop intrathoracic progression in the first year after diagnosis [13]. Some studies suggested that intrathoracic control might be beneficial for the patients in terms of local control (LC) and OS [14–16]. Absence of toxic effects on intrathoracic organs associated with cisplatin-etoposide (EP) regimen over the older doxorubicin-based [17] or cyclophosphamide-based [18] regimens also allow for the use of thoracic radiotherapy (TRT) concurrently. Chemotherapy of EP regimen combined with concurrent TRT is considered the standard of care for patients with limited-stage small cell lung cancer (LS-SCLC) [19]. But controversy remains as to the role of consolidative TRT in ES-SCLC.

The objective of this study was to conduct a systematic review of the published literature to identify the role of TRT in patients with ES-SCLC and to explore the feasible way to deliver TRT regarding the radiation dose, fraction, target volume, and the optimal timing of chemoradiotherapy. Involved studies about TRT for ES-SCLC are summarized in Table 1 [14–16, 20–30].

**Table 1:** TRT regimens for ES-SCLC in different trials

Study	Country	Study type	Patients included*	TRT dose	PCI dose	timing of TRT with CT	Target of TRT	OS			PFS			LC rate			Toxicity of TRT
								TRT	No TRT	P	TRT	No TRT	P	TRT	No TRT	P	
Livingston et al.,1978[20]	USA	Prospective Study	250	3000 rad (cobalt-60) /10f daily in 2w	3000 rad (cobalt-60) /10f daily in 2w	Sequential	The anterior chest portal: supraclavicular LNs bilaterally, mediastinum, and primary lesion as seen on the most recent chest roentgenogram. The posterior portal was designed to oppose the mediastinum and the primary lesion included in the anterior portal.	MST 25w, 1-y OS 15-25%	MST 15-38w, 1-y OS 7-25%	NA	NA	NA	NA	NA	NA	NA	NA
Dillman et al.,1982[21]	USA	Prospective Study	29	4000rad/20f in 2 split courses	2000rad/10f	Concomitant	Entire pre-treatment primary tumor plus 1cm; Ipsilateral hilar and mediastinal LNs, clinically involved supraclavicular LNs	MST 42w	No	No	No	No	No	LRR 6/10	No	No	Mild RIE(2), RIP(1)
Nou E et al.,1988[22]	Sweden	RCT	54	40Gy/20f in 4w	No	Sequential	Primary tumor plus 1- 2cm margin and adjacent mediastinum. No tumor remained: former primary tumor area plus a 2-cm margin and the adjacent mediastinum.	MST 7.6m	MST 9.2m	0.8 < P < 0.9	NA	NA	NA	LRR 15/28	LRR 16/26	NA	No
Beith et al.,1996[23]	Australia	RCT	117	39.6Gy/18f/2.2Gy in 2 split courses	39.6Gy/18f/2.2Gy in 2 split courses.	Concomitant	Primary tumor, mediastinum	MST 42.5w; 2-y OS 9%(11/117)	No	No	NA	No	No	NA	No	No	No severe TRT induced toxicity
Bonner et al.,1995[24]	USA	Phase I/II non-RCT	19	20Gy/5f; hemibody RT also given	17Gy/5f	Concomitant	Ipsilateral hilar, bilateral mediastinal, ipsilateral supraclavicular LNs and post-CT tumor	MST 11.5 m; 2-y OS,25%; 5-y OS, 16%	No	No	5-y PFS,27%	No	No	NA	No	No	grades 3 RIE: 1/19;grade 1-2 RIP:2/19
Jeremic et al.,1999[25]	Germany	Prospective RCT	206	54Gy/36f in 18d	25Gy/10f	Concomitant or sequential	All gross disease and ipsilateral hilum with a 2-cm margin and the entire mediastinum with a 1-cm margin, both supraclavicular fossae	MST 17.0m; 2-y OS, 38%; 5-y OS,9.1%	MST 11.0m; 2-y OS,28%;5-y OS,3.7%	0.041	Median PFS 13m,1-y PFS 5%, 5-y PFS 9,1%	Median PFS 9m, 1-y PFS 41%, 5-y PFS 1.9%	0.045	median LRFS 30m; 1-y LRR, 20%,5-y LRR, 44/55(80%),	median LR FS 22m; 1-y LRR,40%; 5-y LRR,50/54(91.9%),	0.062	grades 3-5 RIE: 42/55; grade 3-5 RIP bronchopulmonary: 8/55
Zhu et al.,2011[14]	China	Retrospective study	119	40-60Gy /1.8-2.0 Gy	Not routine	Concomitant	Primary tumor and the positive LN with a short-axis dimension> 1cm on CT scans	MST 17.0m; 2–y OS,35%; 5-y OS,7.1%	MST 9.3ms; 2–y OS,17%; 5-y OS, 5.1%,	0.014	median PFS 10.0m; 2-y PFS, 12.6%; 5-y PFS, 6.3%	median PFS 6.2m; 2-y PFS,7.2%; 5-y PFS,5.4%	0.0005	19/60	31/59	0.05	Grades 2 RIE, 8/60; grades 3 RIE, 5/60; grades 2 RIP 3/60; grades 3-5 RIP, 2/60
Giuliani et al.2011[15]	Canada	Retrospective study	215	45Gy/30f twice daily, 40Gy/15f,36Gy/12f daily	25Gy/10f (8 patients)	Concomitant or sequential	NA	MST 14.0m; 1-y OS, 58%;2-y OS, 14%	No	No	median PFS 9m; 1-y PFS ,26%;2-y PFS, 0%	No	No	1-y LRR,26%; 2-y LR,39%	No	No	Grades 2 RIE, 2/215
Yee et al.,2012[26]	Canada	Prospective Phase II non-RCT	32	40Gy/15f daily	25Gy/10f	Sequential	Post-CT intrathoracic disease visible on planning CT scan (primary tumor and abnormally enlarged regional LN >1.0 cm)	MST 8.3m; 1-y OS, 2/32; 2-y OS 0%	No	No	median DFS 4.2m; 1-y DFS 10/32; 2-y DFS 0%	No	No	16/32 intrathoracic recurrences	No	No	Grade 2 RIE: 18/32
Slotman et al.,2015[16]	Netherland,UK, Norway, Belgium.	Phase III RCT	495	30Gy/10f	20Gy/5f, 25Gy/10f, or 30Gy/10f, 30Gy/12f, 30Gy/15f	sequential	Post-CT volume with a 15mm margin	MST 8.0m; 1 –y OS,33%; 2-y OS,13%	MST 8.0m; 1-y OS,28%; 2-y OS,3%	1-y P=0.066; 2-yP=0.004	median PFS 4.0m; 6-m PFS 24%	median PFS 3.0m; 6-m PFS=20%	0.001	108/247(43.7%)	198/248(79.8%)	<0·0001	no severe toxic effects
RTOG0937,2015[27,28]	USA,Cananda	Phase II RCT	86	45Gy/15f,30-40Gy/10f	25Gy/10f	No concurrent CT	Original primary disease and involved regional lymphatics.	NA	NA	NA	NA	NA	NA	NA	NA	NA	deaths: 23/39; grades 4-5 toxicities, 7/39
Qin et al.,2016[29]	China	Retrospective study	94	40-60 Gy/ 1.8-2.0Gy daily	NA	concomitant or sequential	Chest lesions, mediastinal, and supraclavicular LNs	MST 13.0m; 1-y OS,56.3%	MST 9.0m; 1-y OS, 30.6%	0.006	median PFS 9.0 m	median PFS 6.0 m	0.018	NA	NA	NA	grades 3-4 RIE, 1/32; grades 3-4 RIP, 1/32
Luan et.,2015[30]	China	Retrospective study	165	40-62Gy, 1.5Gy/f twice or 2Gy/f daily	30Gy(5 patients)	sequential	Post-CT: CR, tumor bed and the locations of the positive LN;SD, primary tumor and the positive LNs; PD, primary tumor, positive LNs and the new lesions	MST 18m; 2-y OS, 35.3%; 5-y OS, 2.4%	MST 12m; 2-y OS,14.5; 5-y OS, 2.4%	0.033	median PFS 9m;1-y PFS,35.4%,2-y PFS,6.0%	median PFS 6m;1-y PFS,20.5%,2-y PFS,6.0%	0.011	(42/82)51.2%	(60/83)72%	0.006	grade2 RIE:2/82;grade 2 RIP:2/82; grade3 RIP: 1/82

TRT, thoracic radiotherapy; ES-SCLC, extensive stage small cell lung cancer; OS, overall survival; PCI, prophylactic cranial irradiation; CT, chemotherapy; OS, overall survival; PFS, progression free survival; LC, local control; f, fraction; w, weeks; MST, median survival time; NA, no available; LN, lymph node; LRR, local relapse rate; RIE, radiation induced esophagitis; RIP, radiation induced pneumonitis; RCT, randomized controlled trial; y, year; LRFS, local relapse free survival; GTV, gross tumor volume; CR, complete response; SD, stable disease; PD, progressive disease

*, “Patients included” were confined to extensive stage small cell lung cancer patients who were enrolled into the final analysis in each study

## THE THEORETICAL FOUNDATION OF TRT IN ES-SCLC

### Increase of local-regional and distant control

Pushed by fears of such a high local regional (LR) failure rate of ES-SCLC, lots of professors have been working on the additive effect of TRT beyond chemotherapy alone for decades. Previous studies conducted in 1970s-1980s failed to observe an improvement in the durability of complete response (CR) and prevention of relapse in regions where disease had been previously identified [20–22]. But recent studies have found promising results with the incorporation of TRT. Professor Meredith et al conducted a retrospective review in a total of 19 patients with ES-SCLC receiving ≥30Gy consolidative TRT sequentially or concurrently with chemotherapy [15]. The cumulative LR failure rates were 26% and 39% at 1 and 2 years respectively, while the median progression free survival (PFS) was 9 months, with a 1-year and 2-year PFS of 26% and 0%, a 1-year and 2-year OS of 58% and 14%, respectively. In contrast, for patients treated with chemotherapy alone who achieved CR at local and complete/partial response (CR/CR) at distant levels, the 1-year and 2-year LR failure rates were 40% and 51% respectively in a previous study [25]. It has been reported that the most common site of progression after chemotherapy alone was the lung, with approximately 30% patients developing lung metastases [6]. The chest irradiation in extensive disease small cell lung cancer (CREST) trial conducted by Dutch Lung Cancer Study Group revealed a nearly 50% reduction in the risk of intrathoracic progression (*P* < 0.001) with the post-chemotherapy (post-CT) TRT for ES-SCLC patients [16]. Thus, similar to LS-SCLC, the consolidative TRT also shows a great potential to bring a high local-regional control and eventually lower the distant metastases.

### Prolonging of survival time

**Figure 1:**
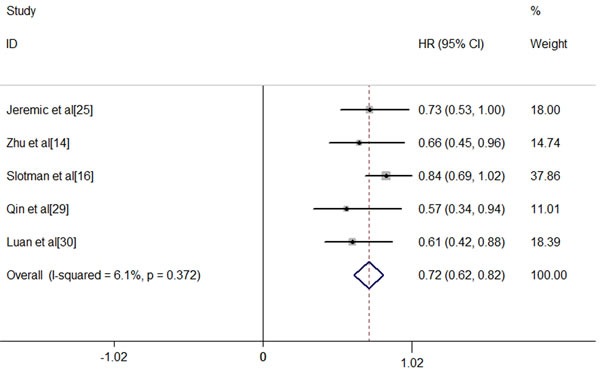
Meta-analysis of OS between ES-SCLC patients receiving TRT or not The addition of TRT was associated with a significant improvement in OS (fixed-effects model HR, 0.72; 95% CI, 0.62-0.82; *P* < .0001). Heterogeneity testing was negative (Q = 4.26, df = 4, *P* = .372, I^2^ = 6.1%).

**Figure 2:**
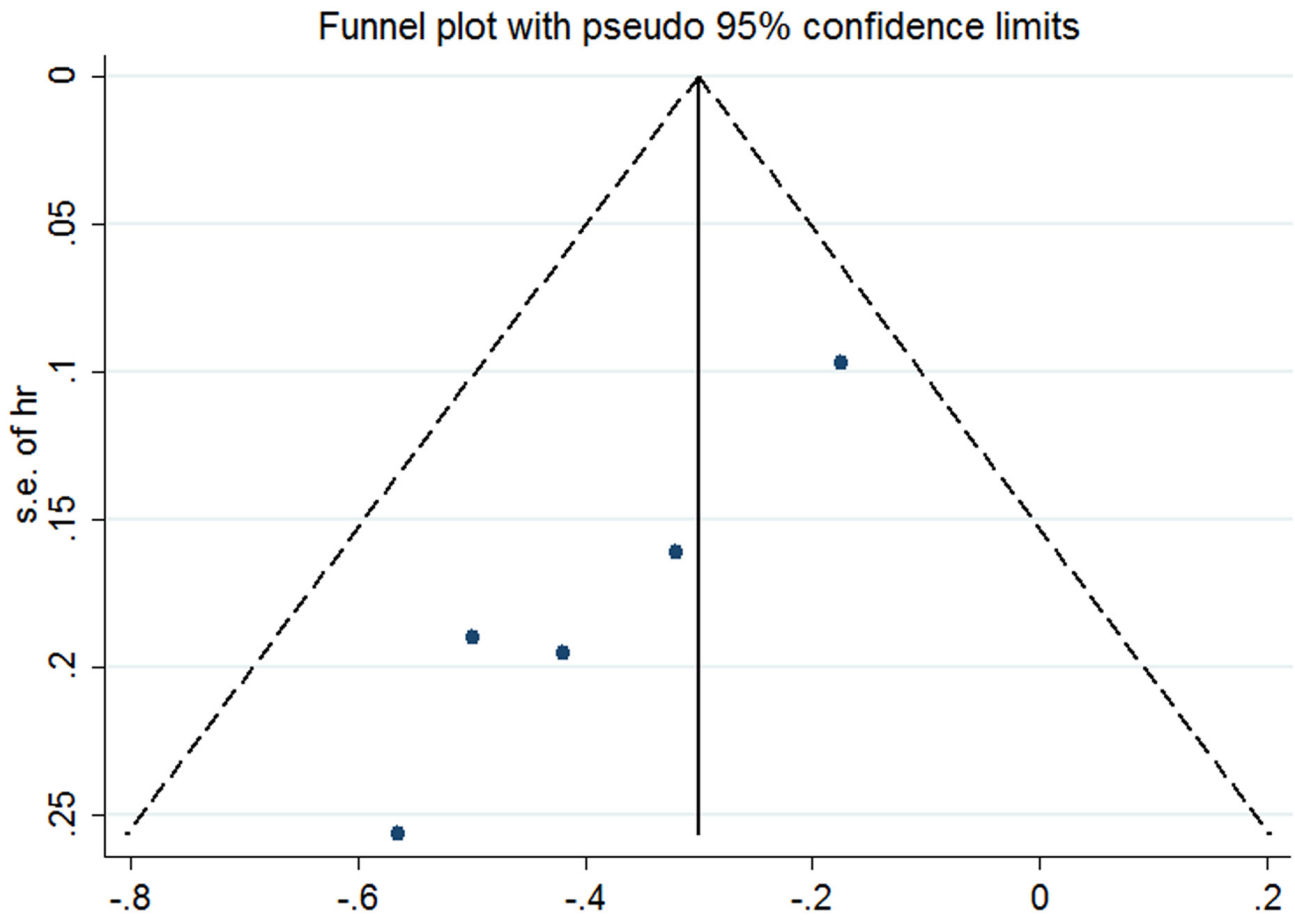
Funnel plot of included studies According to the funnel plot, no significant asymmetry was detected for our outcome.

For ES-SCLC patients, the median natural survival is only 2-4 months [31] and there are virtually no long-term survivors [32]. Combination chemotherapy of etoposide and platinum has improved short-term survival, but long-term survival remains frustrating. The 2-year survival rate among patients with ES-SCLC has risen by only 3.1% from 1.5% in 1973 to 4.6% in 2000 [33]. In 1999, Jeremic et al published the results of a study in which patients with ES-SCLC achieving extrathoracic CR after chemotherapy and either CR or PR inside the thorax were randomized into two groups: prophylactic cranial irradiation (PCI) plus chemotherapy group *versus* PCI, TRT plus additional chemotherapy group. The eventual data found a significantly improved survival with the use of TRT [25]. Recent results from the CREST trial have given rise to a large scale worldwide debates [16]. The study showed that in ES-SCLC patients with any response after chemotherapy, TRT led to a significant improvement in PFS (*P* < 0.001). Although the 1 year-survival difference between two groups (TRT arm 33% *vs*. no TRT arm 28%) failed to reach the expected 10%, a significant difference was observed in 2-years OS (TRT 13% *vs*. no TRT3%). Meta-analysis of Jeremic ‘s study and the CREST study further addressed the value of TRT in ES-SCLC patients receiving EP chemotherapy. Final result indicated that the use of TRT was associated with improvement in both OS and PFS with hazard ratios(HRs) of 0.81(95% confidence interval [CI], 0.69-0.96) and 0.74(95%CI, 0.64-0.87), respectively [34]. This remarkable outcome might result from more chances for patients to receive salvage treatment after failure to first-line therapy. To our knowledge, there have been 7 studies comparing the OS of ES-SCLC patients receiving TRT or not [14, 16, 20, 22, 25, 29, 30], among of which only 5 studies have available data [14, 16, 25, 29, 30]. After a comprehensive and quantitative assessment, we carried out a meta-analysis of these 5 studies and found a significant relationship between TRT and OS. The addition of TRT was associated with a significant improvement in OS (fixed-effects model HR, 0.72; 95% CI, 0.62-0.82; *P* < .0001). Heterogeneity testing was negative (Q = 4.26, df = 4, *P* = .372, I^2^ = 6.1%), and the OS result remained significant in the sensitivity analysis (Figure 1). We had applied sensitive search strategies and rigorous inclusion criteria to minimize the potential publication bias. According to the funnel plot, no significant asymmetry was detected for our outcome (Figure 2).

Similar to the criticism as to the role of PCI for SCLC patients, what should be noted was that treatment for progressive disease could have affected the eventual outcomes: whether the small survival benefit was attributed only to TRT or to the increasing chances of those patients at the time of disease progression who remained in a better shape and fitter for receiving more second-line or third-line chemotherapy.

### Benefit of life quality

Apart from survival benefit, there likely remains value in attaining improved LC in the chest from the quality of life perspective. Post-CT chest recurrences in ES-SCLC patients occur commonly and sometimes lead to some distressing symptoms ranging from cough, wheezing, shortness of breath, orthopnea, hemoptysis and pain to dyspnea, dysphagia, superior vena cava obstruction syndrome, which might become severe enough to become life threatening. Professor Don Yee ever conducted a study to evaluate 32 ES-SCLC patients who attaining an objective response to chemotherapy and tried to define the rate of symptomatic chest failures after undergoing post-CT consolidation TRT [26]. With a median follow-up time of 21.8 months, of all of the 19 intrathoracic recurrences, only 5 were symptomatic. Moreover, just 7/16 of intrathoracic recurrences were in the irradiated region. This indicated that consolidation TRT could not only provide a good LC but also an excellent symptomatic control in the irradiated chest region for ES-SCLC patients.

## THE CURRENT IMPLEMENTATION PATTERN OF TRT

### The dose of the TRT regimen

As we all know, controversy has long persisted over the optimal dose and schedule of chest RT in SCLC patients. For LS-SCLC, twice daily therapy of 45 grays(Gy)/30fraction(f) in 3 weeks is superior to once-daily therapy of 45 Gy/25f in 5 weeks [19]. When using once-daily RT, higher doses of 60-70Gy is recommended by National Comprehensive Cancer Network (NCCN) guideline [35]. The randomized trial CALGB30610/ Radiation Therapy Oncology Group (RTOG) 0538 is undergoing to compare the 45 Gy twice daily in 3 weeks to 70Gy once daily in 7 weeks [36]. A systematic study of 19 trials showed that increased biological effective dose (BED) was associated with prolonged survival and decreased LR relapse rate in LS-SCLC patients in a setting of combined chemoradiotherapy, which indicated the potential value of RT dose escalation strategy over a limited time frame. For ES-SCLC patients, in Jeremic's published trial, the RT regimen of 54 Gy in 38 fractions over 18 days was delivered with concurrent EP chemotherapy [25]. While in the CREST study, TRT was delivered to a dose of 30 Gy in 10 fractions [16]. But the relative high intrathoracic failure rate of 42% suggested that the intensity of 30Gy/10f regimen might be too weak to eliminate all the minimal residual diseases. In another prospective non-randomized phase II study, subsequent consolidative TRT following four cycles of platinum-based chemotherapy, delivered as 40 Gy in 15 daily fractions was well tolerated [26]. RTOG 0937 used a more aggressive hypofractionated regimen, 45 Gy in 15 fractions or 40 Gy in 10 fractions, but unfortunately the study was closed early due to futility leaving no mature results [27, 28]. In the University of Texas M D Anderson Cancer Center (MDACC), standard TRT of 45Gy/15f is delivered to the intrathoracic residual lesions for patients who achieved extrathoracic CR after 4-6 cycles of chemotherapy. For cases with extensive metastases who achieving CR or PR after chemotherapy, only a palliative chest radiotherapy of 30Gy/10f is needed as long as systemic condition permits. For Chinese population, Zhu et al ever retrospectively reviewed the records of 119 ES-SCLC patients, of whom the total TRT dose ranged from 40 to 60Gy at 1.8 to 2.0 Gy per fraction for 5 days weekly [14]. They found that the irradiation ≥50Gy were received by more than half of the patients included (76.7%) at an expense of low level toxicity (Table 1). To sum up, all the doses given above was well tolerated and the standard of TRT dose for ES-SCLC has reached no consensus. It all depends on the doctors’ choice in different institution.

### Radiotherapy in different types of dose-fractionation schedule

As we mentioned earlier, for LS-SCLC patients, the accelerated hyperfraction radiotherapy regimen of twice-daily schedule produced a survival advantage compared with once-daily program. The Eastern Cooperative Oncology Group/ RTOG compared once-daily (QD TRT) to twice-daily thoracic radiotherapy (BID TRT) with concurrent chemotherapy of EP in 417 LS-SCLC patients with a total dose of 45Gy. Both the median survival (20 versus 19months) and 5-year survival rate (23% *versus* 16%) were better for twice-daily group (*P* = 0.04). [19] But there appears to have been a significant criticism for the non-equivalent biological doses of radiation in the 2 arms of this trial. In light of this ongoing trial are evaluating BEDs of 45Gy delivered twice daily *versus* 60 to 70 Gy delivered once daily.

The translation of QD to BID irradiation regimen also has advantageous biological basis. Short-course RT could help avoid the appearance of accelerate repopulation for SCLC cell which has a relatively short potential doubling time of about 3-5 days. Furthermore, *in vitro*, lack of shoulder in the dose-response curves for SCLC lines indicated that small cells could be killed even at relatively low doses per fraction, implying that radiotherapy with multiple small fractions could kill small-cell cancer at expense of minor permanent damage to ambient normal tissues and decreased risks of late effects [37].

Xu et al have ever evaluated the correlation between different fractionation schedule of radiotherapy with the LC rates and OS in Chinese ES-SCLC patients. In the end, no significant difference was observed in 2-year OS, PFS, or LC rates between hypo-fractionation and conventional fractionation group (35% vs. 26%, *P* = 0.886; 18% vs. 16%, *P* = 0.560; 67% vs. 36%, *P* = 0.159). So they think the hypofractionated radiotherapy has similar efficacy but substantially shortened radiation time compared with conventionally fractionated radiotherapy [38]. Another concern regarding hyperfractionation is that BID TRT is technically challenging for patients with bilateral mediastinal lesions. Thus, patients selected for combined modality treatment which incorporates BID TRT must have excellent performance status and baseline pulmonary function. Overall, both QD and BID TRT are optional before much more evidence-based results have been carried out.

### The target volume of the TRT regimen

Studies in the last century always chose to delineate an extensive target volume in order to involve all the micro sub-clinical tumors and ultimately eliminate them as far as possible. Today, most of radiation oncologists tend to outline a limited involved target volume according to the post chemotherapy images. In MDACC, the irradiation target volume was also determined based on the post-CT evaluation. They think TRT of 45Gy/15f should be given to the residual lesions for patients achieving post-CT PR. If no lung lesions left, only the initially involved LNs receive RT while no radiation is given to the primary lung tumor due to the difficulty in finding the exact location of possible residual subclinical lesions. Patients with multi-metastases would receive only a palliative radiation of 30Gy/10f for the intrathoracic tumor. Similar guidelines are implemented in a large majority of Chinese medical institutions including our department.

### Timing of radiotherapy

There has been much debate about the optimal timing of TRT in SCLC patients. Split-course radiotherapy has been abandoned due to the inefficacy caused by tumor regrowth between courses. Various studies were performed in LS-SCLC patients to solve the question of when TRT should be implemented. An early meta-analysis in 1992 identified no differences regarding the timing of thoracic radiotherapy and chemotherapy in LS-SCLC cases [39]. But the severe toxicity associated with early chemotherapy regimens (alkylator/anthracycline-based regimens) might have affected the results. Recently, Ruysscher et al performed an individual patient data meta-analysis of 2,305 patients in 9 randomized trials to compare earlier *versus* later radiotherapy, or shorter *vs*. longer radiotherapy duration, as defined in each trial. When all trials were analyzed together, “earlier or shorter” *vs*. “later or longer” thoracic radiotherapy did not affect OS. But the “between-arm” chemotherapy compliance (number of cycles actually given) is the leading cause of between-trial heterogeneity. Thus, in the subset data analysis among trials with a similar compliance with chemotherapy in both arms as defined, the hazard ratio (HR) for OS was significantly in favor of “earlier or shorter” radiotherapy (HR 0.79, 95% CI 0.69-0.91). The absolute gain between two arms in 3-year and 5-year OS rate were 5.7% and 7.7%, respectively, while in 3-year and 5-year PFS rate were 6.3% and 5.6%. However, “earlier or shorter” thoracic radiotherapy was associated with a relatively higher incidence of acute esophagitis (14% vs.8%) [40]. So, the NCCN guidelines recommend that TRT should be initiated during the first or second cycles of chemotherapy in cases of LS-SCLC whether delivered twice or daily per day [35, 41]. But evidence to the contrary also exit. The randomized phase III trial in South Korea demonstrated that late TRT was not inferior to early TRT in terms of the remission rates, OS, PFS, treatment failure patterns, and was superior as to the grade 3 or 4 esophagitis/ pneumonitis (no significant difference) [42].

As to the optimal timing of TRT in ES-SCLC patients, not much evidence existed due to the rare studies designed especially for these specific population. Considering the relatively higher incidence of irradiation-induced toxicity, a large majority of institutions delivery TRT sequential to chemotherapy. In MDACC, standard treatment of TRT with or without PCI was delivered to patients who achieved CR or PR after 4-6 cycles of systematic chemotherapy. If patients cannot tolerate simultaneous PCI, they could receive TRT before PCI.

### Treatment-related toxicities

Use of TRT brought not only improvements in survival and tumor control, but also a relatively higher treatment toxicity. However, the TRT appears to be well tolerated. Meta-analysis indicated that use of TRT did not increase the bronchopulmonary toxicity (≥grade 3) (TRT 2% vs no TRT 1.7%). Esophageal toxicity (≥grade 3) remains uncommon and happened in only 6.6% of all the included patients, most of whom receiving a TRT dose of 30Gy/10f [34]. Generally, concern is frequently expressed about the “toxicities” of the addition of radiation to chest, ignoring the significant toxicities of chemotherapy even the outcomes of no treatment. In Jeremic's study, though more patients in TRT plus chemotherapy arm experienced bronchopulmonary and esophageal toxicities, grade 3±4 acute toxic events appeared less frequent (88/55) in TRT plus chemotherapy arm compared with the chemotherapy alone arm(145/54) (P = 0.0000). In the end, no significant difference was found in either late grade 3 (2/55 *vs*. 0/54, *P* = 0.16), late grade 4 (1/55 *vs*. 0/54, *P* = 0.32), or combined late grade 3+4 (3/55 *vs*. 0/54, *P* = 0.082) toxicity between these two groups. Moreover, different radiation therapy techniques available should also be considered. With the development of therapeutic devices, more patients have a chance to receive three dimension conformal radiotherapy or intensity modulated radiation therapy rather than two dimensional treatment, which help reduce the radiation dose to normal tissues around. Review of the past studies could find that most studies with TRT-induced toxicities were delivered in twice daily schedule (Table 1). We could speculate that daily irradiation regimen in a relative longer treatment time with higher dose might lead to a better outcome with less toxic incidences.

A diagram was drawn to illustrate the recommended way to administrate TRT for selected patients (Table 2).

**Table 2:** Recommended TRT regimens for ES-SCLC

	Recommended therapy
Irradiation dose	30Gy/10f^a^, 40 - 60Gy
Dose- fraction schedule	Once-daily or twice-daily
Radiation field	Post-chemotherapy PR: residual lung lesions + the initially involved lymph nodes Post-chemotherapy CR: the initially involved lymph nodes
Timing of radiation	After 4-6 cycles of systematic chemotherapy
Possible suitable population of TRT	Patients with a good or partial response after chemotherapy. Patients who have minimal burden of metastatic disease or a good control in other metastases Patients with favorable prognostic factors (eg, limited metastases, early disease stage, excellent performance status)

TRT, thoracic radiotherapy; ES-SCLC, extensive stage small cell lung cancer; CR, complete response; PR, partial response

^a^ The palliative chest radiotherapy of 30Gy/10f is only available for cases with multi-metastases who achieving CR or PR after chemotherapy as long as systemic condition permits.

## WHAT IS THE POSSIBLE SUITABLE POPULATION OF TRT TREATMENT?

Like other medical therapies, not all the ES-SCLC patients could benefit from consolidative TRT treatment. It is clear that we need to identify those patients benefiting most from TRT, from the prospective of quality of life, survival and treatment toxicity. The CREST study has performed additional analysis to identify the possible suitable population of TRT, and found that TRT led to a significant difference in OS and PFS in a particular group of patients who had residual intrathoracic disease after chemotherapy [16]. In patients who achieved a complete intrathoracic response, no benefit of TRT was observed. Based on the additional analysis highlighted above, they concluded that TRT should be offered to patients with a good or partial response after chemotherapy, but not those without residual disease in the thorax [16].

As we all know, according to VALSG staging system [1], ES-SCLC includes obviously diverse and “extensive” clinical situation ranging from “limited” extensive disease of locally advanced thoracic disease that cannot be encompassed in a typical radiation portal to grossly metastatic disease. The metastatic disease which has a bad control in extrathoracic lesions might not benefit a lot from TRT, whereas the associated treatment toxicity might increase. Just like what we have observed in LS-SCLC patients, the consolidative TRT only improve OS and LC in a highly selected subset of ES-SCLC patients who have minimal burden of metastatic disease and are suitable for aggressive management of thoracic radiotherapy in addition to chemotherapy. At the same time, the role of TRT in a subgroup of patients with less favorable prognostic factors (eg. multiple metastatic sites, advanced disease stage, poor performance status, elevated serum level of lactate dehydrogenase [43]) requires further studies to explore. Based on the available evidence, possible suitable population of TRT treatment were outlined in Table 2.

## NOVEL DEVELOPMENTS IN ES-SCLC

PCI has been demonstrated to increase survival after platinum-based chemotherapy in ES-SCLC patients with no evidence of brain metastases at staging. Furthermore, the study also conducted by Slotman concluded that PCI could bring about a significantly improved survival and a significant reduction in brain metastases rate with limited adverse effect on global health status as well as on functioning scores [44]. So investigation of the combination of PCI and TRT in patients with ES-SCLC without brain metastasis was one such initiative. Among of them, RTOG0937 eventually failed to demonstrate the survival benefit of PCI plus consolidative radiation to the chest and oligometastatic sites (up to 4 sites of extracranial metastasis). But the preliminary analysis results reported in the 2016 American Society for Radiation Oncology (ASTRO) revealed that consolidative radiation therapy to the thorax and extracranial metastases delayed progression of disease. Toxicity associated with the combination of chemotherapy and radiation to both the chest and oligometastatic sites might have mitigated the survival benefit [45]. We could speculate that aggressive management with chemotherapy and consolidative TRT as well as PCI are also only applicable to a highly selected subset of ES-SCLC patients who have minimal burden of metastatic disease. In addition, considering the poor survival of ES-SCLC patients, short and relatively low-dose fractionation schemes were recommended.

Combination chemotherapy of platinum and etoposide remains the standard of care for ES-SCLC patients [46]. However, more evidence from high quality-control, large sample sized, and prospective studies are in need to corroborate rationality of this protocol. A randomized trial in Japan (JCOG9511) compared the combination of cisplatin with either etoposide or irinotecan (IP) in ES-SCLC demonstrated that the IP was superior to EP (median OS, 12.8 months *vs*. 9.4; 2-year survival rate 19.5% *vs*. 5.2%) [47]. However, evidence from three confirmatory Western studies failed to corroborate the impressive survival average over one year in the Japanese study [48–50]. These may result from pharmacogenomic differences between Japanese and Western populations in the metabolism of irinotecan. So the couplet of etoposide and cisplatin remains the standard first-line treatment for ES-SCLC. More researches in lab and clinic would continue to identify more chemotherapeutic drugs with a longer duration of treatment effectiveness.

In systemic diseases like ES-SCLC, the most impressive advances recently have been in the use of targeted drugs. Though lots of targeted drugs have failed to reach the scheduled goal, more exploratory researches are still under way. The Phase I trial, E2511, has already demonstrated the safety of combining EP with veliparib- a poly ADP-ribose polymerase (PARP) inhibitor which prevents single-strand DNA repair in previously untreated ES-SCLC [51]. A follow-up Phase II study of this combination and other additional studies with other PARP inhibitors and DNA damage response modulators in ES-SCLC are underway. Furthermore, the advent of high-sensitive, next-generation DNA sequencing technologies has revealed that SCLC is characterized by a high mutational burden, which may help identify more targetable molecular abnormalities and develop novel targeted therapies.

Development of diagnostic imaging also contributes to the treatment of ES-SCLC. Among of them, position-emission tomography (PET) scanning has been able to discern regions of residual viable disease and help outline all regions of residual metabolically active intrathoracic disease after chemotherapy [52]. New biological imaging methodologies, such as PET/CT, magnetic resonance imaging (MRI) and magnetic resonance spectroscopy (MRS) imaging could be used to draw a three-dimensional map of radiobiological relevant parameters to trace the real target volume and make dose-painting possible.

In addition, TRT induced cell death might lead to an immunological anti-tumor response in addition to the local effect which merits further investigation. As we all know, increasing evidence shows that SCLC is immunogenic, supporting the rationale for using immunotherapy in SCLC [53]. The immunotherapeutic agents being investigated recently are focused on antibodies that target the programmed cell death protein-1 (PD-1; nivolumab and pembrolizumab) and cytotoxic T-lymphocyte antigen-4 (CTLA-4; ipilimumab) pathways [54]. In the phase I KEYNOTE-028 study, for patients with PD-L1-positive ES-SCLC who did not respond to first-line therapy, pembrolizumab yielded a 29% ORR, with durable responses [55]. Based on the preclinical and clinical findings of that the certain chemotherapeutic agents could augment the activity of immunotherapy, some ongoing trials are investigating the combination use of immunotherapy and chemotherapy. The significant trend toward improved ir(immune-related)-PFS and OS have been observed in the phase II trial (NCT00527735) with ipilimumab in combination with paclitaxel and carboplatin [56]. While in the randomized, double-blind phase III study (CA184-156) which evaluated the efficacy and safety of ipilimumab or placebo plus etoposide and platinum in patients with newly diagnosed ES-SCLC, the addition of ipilimumab did not result in a statistically significant improvement in OS *versus* chemotherapy alone [57]. The inconsistencies in recent reports indicated the necessity to identify predictive immune-based biomarkers which help select the patients most likely to benefit from immunotherapy. Moreover, there are a lot of questions to be answered before the immunotherapy is established as the standard treatment, including combination immunotherapy, or combination of immunotherapy with chemotherapy/ radiotherapy, the timing of these therapies.

## CONCLUSIONS

Various studies suggest a survival benefit from consolidative TRT but variability in patient selection, staging studies, and radiation therapy technique makes the application of these results problematic to be considered as a new standard of care at present. More prospective large-sample-size randomized controlled trials are in need to determine the standard practical recommendations of TRT in ES-SCLC and explore more novel ways to help patients live better, less painful, prolonged survival.
